# Optimisation of AgNP Synthesis in the Production and Modification of Antibacterial Cellulose Fibres

**DOI:** 10.3390/ma14154126

**Published:** 2021-07-24

**Authors:** Emilia Smiechowicz, Barbara Niekraszewicz, Piotr Kulpinski

**Affiliations:** Department of Mechanical Engineering, Informatics and Chemistry of Polymer Materials, Faculty of Material Technologies and Textile Design, Lodz University of Technology, Zeromskiego 116, 90-924 Lodz, Poland; barbara.niekraszewicz@p.lodz.pl (B.N.); piotr.kulpinski@p.lodz.pl (P.K.)

**Keywords:** nanocomposite fibres, lyocell, antibacterial cellulose fibres, silver nanoparticles, N-methylmorpholine-N-oxide (NMMO)

## Abstract

The main aim of the presented research is to determine the optimal conditions for the production of silver nanoparticles (AgNPs) in N-methylmorpholine-N-oxide (NMMO), which will potentially allow to obtain small nanoparticles with uniform diameter distribution. In this paper, NMMO is used in the fibre production process, both as a direct cellulose solvent and as an Ag+ reducing system. From an industrial point of view, this method is very promising because it allows to reduce the amount of used chemicals. The UV/Vis, DLS and TEM analysis proved that the synthesis temperature and time could play a key role in nanoparticle growth control in NMMO. It was found that the optimal conditions for AgNPs synthesis are 100 °C and 0.33 h. The estimations of the antibacterial activity of the fibres were completed. The applied AgNPs synthesis conditions allow to obtain antibacterial fibres with a wide range of applications, mainly in medicine.

## 1. Introduction

Silver nanoparticles (AgNPs) are among the most popular nanoparticles created using physical, chemical and biological methods [1,2,3,4,5]. Khodashenas and Ghorbani analysed various methods of synthesising silver nanoparticles for obtaining different shapes and particle sizes. They concluded that cubic and spherical silver nanoparticles can be obtained by controlled synthesis through chemical, physical or biological methods. Chemical and physical methods play the most important role, as currently it is very difficult to carry out shape-controlled syntheses using biological methods [6]. The generated nanoparticles have suitable chemical and physical properties required in the selected application, namely in medicine, composites or clothes. For this reason, it is advisable to select the best method for the synthesis of nanoparticles, which will allow to obtain nanoparticles with the suitable morphology for further application.

The basic parameters of the generated AgNPs strongly depend on their synthesis conditions. Certain factors of nanoparticles synthesis have a decisive influence on the morphologies of the generated nanoparticles. Recent research shows that the concentration to volume ratio of reacting substances, their reaction time, temperature and pH are the most important factors that influence the synthesis rate. It is possible to plan the morphology of generated nanoparticles by the appropriate selection and control of the mentioned parameters. Jiang XC et al. investigated the role of the temperature of the reaction in the formation and growth of AgNPs [7]. They used two to three reducing agents at the same time in a synergetic reduction. It was shown that the shape and size of nanoparticles can be controlled by the reaction synthesis temperature, especially for silver nanoplates. It was shown that low temperatures (~0 °C) can slow down significantly the formation and growth reaction, which usually takes tens of hours to complete. The reaction rate increases between 17 and 55 °C, as does particle size (the edge length of silver nanoplates increases from ~90 to ~180 nm, and the diameter of spheres from 25 to 48 nm).

Amin et al. observed similar trends and showed that as the temperature and the time of “green” synthesis of nanoparticles increases, the speed of the precursor reaction increases as well [8]. The presented research has shown that when using AgNO_3_ with Solanum xanthocarpum extract, the intensity of absorption also increases with the reaction time. While the reaction temperature increases from 25 to 45 °C, the obtained absorbance maximum visibly increases and shifts from 433 to 406 nm at the highest temperature. It turns out, however, that after increasing the temperature to 50 °C, a visible shift in the maximum absorbance to 450 nm takes place, which indicates the presence of larger size nanoparticles or their aggregates in the examined systems.

Dada et al. presented the operational parameters (factors) and characterisation relevant to the synthesis of silver nanoparticles [9]. Among them, temperature and time were characterised. Studies in ultraviolet visible spectroscopy (UV-Vis) and surface plasmon resonance (SPR) for silver nanoparticles have shown that as the temperature increases, the intensity of the plasmon band also increases due to a bathochromic shift. As a result, the mean diameter of silver nanoparticles is reduced. Sometimes, the reaction of synthesising silver nanoparticles is quite fast, initially. It should not be assumed, however, that such a low temperature is optimal for the tested system. Studies have shown that this is rather related to the action of the reducing or stabilising agent. The influence of synthesis time on the formation of silver nanoparticles was also examined using UV-Vis. The spectrum showed that the intensity of the peak increases alongside reaction time. This resulted in the formation of more silver nanoparticles, but when the process was delayed absorption intensity and wavelength decreased.

Qin et al. observed that by increasing the pH from 6 to 11.5, the average size of the generated silver nanoparticles decreased from 73 to 31 nm [10]. It was shown that after heating at 100 °C for 2 h, the shape of the generated particles became more spherical. Kokila et al. generated AgNPs through biosynthesis using Cavendish banana peel extract [11]. It was presented that the absorbance value was increased gradually alongside a pH range from 2 to 8. It was suggested that the rate of synthesis of AgNPs is higher in basic pH than in acidic. This phenomenon can be explained based on the literature [12,13]. The rate of the reaction depends on the ionisation of the functional groups at higher pH.

Yousof et al. investigated the process of obtaining high-yield Ag nanoparticles (AgNPs) from silver salts (AgNO_3_) using the chemical method and a reducing agent (tri-sodium citrate) [14]. The highest-yield AgNP synthesis was carried out with the following parameters: concentration of AgNO_3_—1 mM; initial pH of AgNO_3_—7; concentration of reducing agent—1%; reaction time between precursor and reducing agent—3.5 min; stirring time—15 min. The obtained AgNPs showed good antimicrobial activity.

There are known examples of the production of AgNPs in cellulose materials [15,16]. They relate to the treatment of cotton or cellulose fibres with an AgNO_3_ solution under various conditions. The authors have mainly studied the most favourable conditions for the introduction of the appropriate amount of silver nanoparticles on cellulose fibres to achieve an antibacterial effect for the fibres and/or fabrics. Other examples are the production of silver nanoparticles in cotton fibres and cellulose fibres using the initial activation and swelling of fibres in a NaOH solution [17,18]. This substance opened the surface and internal pores of the fibres allowing penetration of a silver nitrate solution [18] or an AgNO_3_/NH_4_OH solution [17] into the structure of the fibres. The authors point out the great advantage of AgNPs (nanofluidic system) synthesis conducted in this way over the classical method (bulk solution), especially in the field of process control. It should be noted, however, that the experiment was carried out on a small fibre sample (0.5 g) and many compounds were used to perform this modification. The process was multi-stage. On a larger scale or in an industrial application, such a method of synthesis can probably cause technical problems (several stages, additional rinsing processes). Moreover, the durability of such a modification on the fibres is limited. A significant disadvantage of this method is that a large amount of AgNPs is removed during the required rinsing of fibres or textiles.

Our works to date have involved the production of cellulose fibres using silver nanoparticles introduced at the stage of cellulose dissolution. A previous study has shown that the synthesis of AgNPs during the dissolution of cellulose in NMMO is not as effective as the production of AgNPs in NMMO solution in a separate process, and the introduction of AgNPs thus produced into the system [19]. In this process, NMMO plays the role of both an Ag+ reducer and a cellulose solvent, which reduces the number of reagents used. Furthermore, the proposed process does not require the use of stabilising agents. Controlling the synthesis temperature and time achieves high efficiency and obtains nanoparticles with optimal expected parameters. We examined the production of silver nanoparticles using three different methods under varying light conditions (daylight and darkness). We discussed the influence of the morphology of silver nanoparticles on the colour of obtained fibres [20]. Finally, the influence of silver nanoparticle morphology on the antibacterial properties of fibres was presented in our latest publication [21].

The novelty of this work lies in the optimisation of the process of silver nanoparticles synthesis in NMMO in terms of highest possible efficiency (shortest possible AgNP synthesis time, nanoparticles without agglomerates with the best distribution in the fibre matrix), which also affects its cost-efficiency. Optimisation of the AgNP synthesis process in NMMO reduces the costs of the production process of cellulose fibres modified with AgNPs. Additionally, the use of NMMO in the fibre production process, both as a direct cellulose solvent and as an AgNO_3_ reducing system is very promising because it reduces the amount of chemicals used.

The hypothesis put forward in this work is that it is possible to control and program the process of synthesis of silver nanoparticles using NMMO as a reducing system, paying particular attention to the basic parameters of silver nanoparticles generated in fibres, i.e., their diameter and shape, and the size and quantitative characterisation of AgNP aggregates and agglomerates.

The main aim of the presented research is to confirm the hypothesis by estimating the basic conditions of the production of silver nanoparticles in NMMO. Understanding the influence of the conditions of synthesis will allow to control the process in such a way as to obtain high quality cellulose fibres in optimal conditions with uniform distribution in the cellulose matrix and the lowest possible concentration of silver nanoparticles providing good antibacterial properties. The optimal concentration of silver nanoparticles in the fibres matrix was determined on 500 ppm [22]. It is also important that the nanoparticles create as few aggregates and agglomerates as possible. In this study, we discussed the role of temperature and time in the growth of silver nanoparticles and their influence on the basic parameters of the generated nanoparticles. The influence of synthesis temperature and time on the size, shape, as well as distribution of silver nanoparticles in the polymer matrix of cellulose fibres was investigated by a UV-Vis spectrometer, dynamic light scattering (DLS) and transmission electron microscopy (TEM). The fibres have bioactive properties which means that they could find a wide range of application, mainly in medicine.

## 2. Materials and Methods

### 2.1. Materials

Cellulose pulp (PLACETATE, RayonierAdvenced Materials, Montreal, Quebec, Canada) with a polymerisation degree of DP = 1.236 was used in the present study.

A 50% aqueous solution of NMMO (Huntsman Holland BV, Rotterdam, South Holland, The Netherlands) was used as a direct solvent.

Tenox PG (propyl ester of gallic acid) (Sigma^®^, Gillingham, Dorset, UK) was used as an antioxidant.

Silver nitrate (AgNO_3_) (CHEMPUR, Piekary Slaskie, Slaskie, Poland) was used for the generation of metallic silver nanoparticles.

### 2.2. Preparation of the Pre-Incubated Solutions of NMMO-AgNO_3_ and Cellulose Fibres Modified with Silver Nanoparticles

In order to obtain cellulose fibres containing silver nanoparticles, the following procedure was carried out: for the theoretically calculated concentration of silver nanoparticles in the fibres to equal 0.05% (500 ppm). A suitable amount of aqueous solution (0.01 m AgNO_3_) was added to a 50% aqueous solution of NMMO (NMMO-AgNP system). The obtained solution was stored in a dark room at 20, 50 and 70 °C with different reduction times for each test: 12, 24, 48, 72 and 168 h, at 100 °C at 0.33 h (20 min). All syntheses of silver nanoparticles were carried out in the presence of NMMO only (without cellulose pulp).

In the next step, cellulose, antioxidant Tenox PG, the NMMO-AgNP system and additional amount of NMMO (to obtain the desired composition of spinning dope) were mixed. Dissolution was carried out in vacuum (130 hPa) at 110 °C for about 1.5 h. The obtained spinning dope contained 8% of cellulose.

**Scheme 1:**
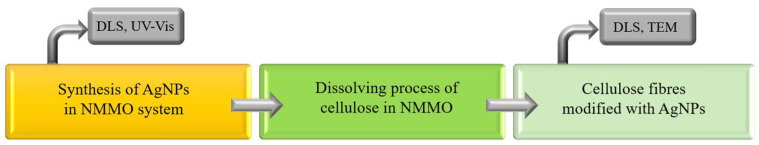
The formation process of the cellulose fibres modified with AgNPs and applied research methods.

Fibres were spun using the dry–wet method on a laboratory spinning machine with a spinneret consisting of 18 orifices. The spinning solution flow rate was fixed at 1 m/min and the temperature of the spinneret during spinning was 115 °C. Fibres were spun into an aqueous spinning bath at a temperature of 20 °C. The fibres were spun at a take-up speed of 50 m/min then washed and dried [19] (Scheme 1).

The abbreviations for the samples used in the present research:

NMMO—50% of water solution of NMMO without modifier

NMMO-Ag/20/12—solution of NMMO with silver nanoparticles, generated at 20 °C, over 12 h

F0—standard cellulose fibres without modifier (unmodified fibre)

F-Ag/20/12—cellulose fibres modified with silver nanoparticles, generated at 20 °C, over 12 h

The used abbreviations consist of three parts: the first part means the type of tested material (solution or fibre), the second refers to the applied synthesis temperature, and the third to the duration of the synthesis.

### 2.3. UV/Vis Measurements

In order to estimate the progress of AgNP formation in pre-incubated NMMO-AgNO_3_ systems UV/Vis spectrophotometry was used. Analysing the obtained UV/Vis spectra, the degree of conversion of silver ions in NMMO was determined, depending on the temperature and time of silver nanoparticles synthesis. Silver nanoparticles have the ability to absorb visible radiation. This is the effect of the surface plasmon resonance phenomenon occurring on them with the maximum in the range from 400 to 500 nm. The wavelength changed depending on the morphology of the generated silver nanoparticles, as well as the presence of their aggregates. The estimation of the progress of the silver nanoparticles synthesis was carried out using Jasco V-570 UV-Vis spectrophotometer (JASCO INTERNATIONAL Co., Tokyo, Japan) in the wavelength range 250–700 nm. Dispersions of nanoparticles in NMMO systems were diluted at a ratio of 1:1 with distilled water. NMMO diluted with distilled water in the same ratio was the reference solution.

### 2.4. Dynamic Light Scattering (DLS)

DLS is a proven non-invasive technique, which can be used to determine the size distribution of small particles in suspension or in solutions of polymers. It can be used to investigate effectively particles of less than 1 nm diameter. The measurements of the size of silver nanoparticles in pre-incubated dispersion were performed at a temperature of 25 °C. The size of nanoparticles, as well as their volume distribution profiles were analysed. The DLS technique and measurement procedure have been described in previous works [19]. The size and volume distribution of silver nanoparticles were determined with the dynamic light scattering technique (DLS) with a PSS Nicomp 380 particle sizer (PSS NIKOMP, Santa Barbara, CA, USA) system. The DLS analysis of the size of silver nanoparticles was done based on Nikomp Distribution with PS Nikomp software’s CW 388 application (v. 1.55).

### 2.5. Transmission Electron Microscopy (TEM)

The morphology of the nanoparticles was estimated using transmission electron microscopy (TEM) TECNAI SuperTWIN FEG (200 kV) (FEI Co., FEI Electron Optics B.V., Eindhoven, Noord-Brabant, The Netherlands). The microstructure of modified fibres was examined in bright and dark fields using a TEM micrograph (P/19/IB-05 3rd edition, 25 July 2003). The presented TEM images included observations of the silver nanoparticles’ distribution in the matrix of cellulose fibres. In order to estimate the distribution size and the shape of silver nanoparticles, the TEM method performed with bright field (BF) (procedure P/19/6 IB-05) and high resolution (HR) techniques was used. The nanoparticle shape and size distribution were assessed by the NIS-ELEMENTS software, and silver nanoparticle size distributions in the fibres were carried out.

### 2.6. Estimation of the Antibacterial Activity of the Fibres

The Japanese Industrial Standard (JIS L 1902: 1998 ‘Testing method for antibacterial of textiles’) was used for the evaluation of the antibacterial efficiency of the modified fibres. The test was performed using the Gram-negative strain of *Escherichia coli* (ATCC 11229) and the Gram-positive strain of *Staphylococcus aureus* (ATCC 6538).

## 3. Results and Discussions

### 3.1. Estimation of AgNP Synthesis Progress

**Figure 1:**
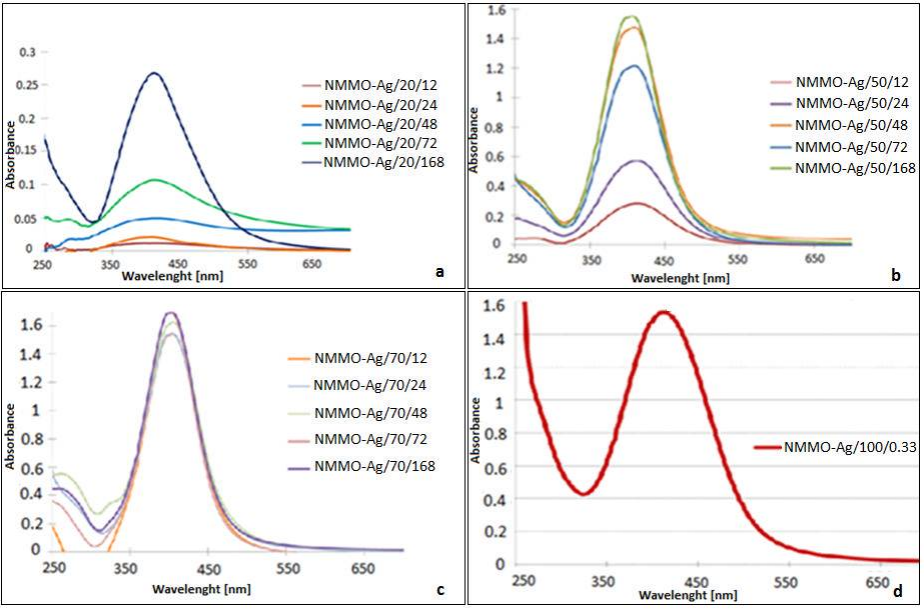
(**a**–**d**) Comparison of the relationship between the absorbance and the wavelength of silver nanoparticles synthesised in NMMO with variable temperatures and times.

**Figure 2:**
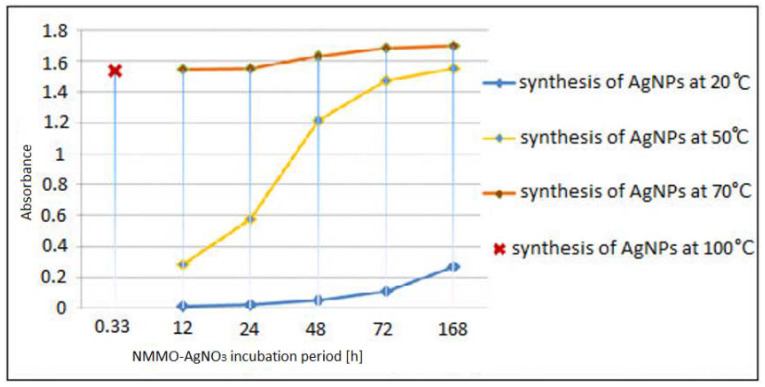
Comparison of absorbance values for silver nanoparticles obtained in NMMO depending on temperature and duration of synthesis.

Spectrophotometric measurements allowed a preliminary characterisation of NMMO—a precursor of silver nanoparticles systems. Based on the obtained UV-Vis spectra of silver nanoparticles generated in NMMO with variable time and temperature, it is possible to estimate the degree of conversion of the silver nanoparticle precursor in NMMO. The width of the obtained spectra allows to assess whether the tested systems modified with nanoparticles are mono- or poly-dispersive. Absorbance measurements as a function of wavelength were made for all NMMO solutions with silver nanoparticles, synthesised with variable temperatures and times (Figure 1a–d). Figure 2 shows the comparison of absorbance values for silver nanoparticles obtained in NMMO depending on temperature and duration of synthesis.

#### 3.1.1. UV-Vis Results of AgNPs Synthesised at 20 °C

Figure 1a shows the relationship between the absorbance and wavelength of silver nanoparticles obtained in NMMO at 20 °C with variable synthesis time.

For the studied NMMO-AgNO_3_ systems, where the synthesis of nanoparticles was carried out at 20 °C for 24–168 h, a change in the maximum absorbance in the wavelength range 411–415 nm is observed depending on the synthesis time (Figure 2). This confirms the presence of silver nanoparticles in the tested solutions. In the case of 12 h synthesis of silver nanoparticles, the reaction progress is very small (absorbance about 0.01). This means that the amount of nanoparticles produced in the system is negligible, and the process of particle synthesis is at the initial stage—in the nucleation phase and slow growth of the nanoparticles. Analysing the obtained spectra, it can be noted that by increasing the synthesis time of silver nanoparticles at 20 °C, the absorbance value increases (Figure 1a). This is the effect of a higher degree of silver nitrate reaction in NMMO, which in turn results in an increase in the number of generated silver nanoparticles in the studied systems. In this case, the synthesis temperature of 20 °C is too low for the nanoparticle precursor to react in NMMO completely, which is confirmed by low absorbance values (from about 0.01 to about 0.3 at the longest synthesis time) (Figure 2). Even the longest duration of synthesis at this temperature is not sufficient to reduce completely silver nitrate in NMMO. Relatively wide peaks in the wavelength range of 340–500 nm (Figure 1a) may indicate the polydispersity of silver nanoparticles generated in the studied systems.

#### 3.1.2. UV-Vis Results of AgNPs Synthesised at 50 °C

Figure 1b shows the relationship between the absorbance and the wavelength of silver nanoparticles obtained in NMMO at 50 °C with different synthesis times (12–168 h).

As can be seen from the graph above, the shortest synthesis time of silver nanoparticles in NMMO (12 h) at 50 °C allows obtaining a comparable degree of conversion of the precursor as observed in the longest time of the synthesis of nanoparticles at 20 °C (absorbance of about 0.25). By increasing the synthesis time, a significant increase of the absorbance value is observed. This is more pronounced for longer reduction times, i.e., from 48 to 168 h (absorbance from about 1.2 to about 1.55, respectively). This means that with the prolongation of the precursor reduction time in NMMO, the amount of silver nanoparticles generated in the systems increases.

#### 3.1.3. UV-Vis Results of AgNPs Synthesised at 70 °C

Figure 1c presents the relationship between the absorbance and the wavelength of silver nanoparticles obtained in NMMO at 70 °C with different synthesis times (12–168 h).

Based on the obtained results it can be noted that the speed of the reaction at 70 °C is much higher than at 50 °C (Figure 1c). It is observed that for the synthesis at 70 °C, the absorbance value increases slightly from about 1.55 for the shortest synthesis times (12 and 24 h) to about 1.7 for longer times (168 h). The absorbance obtained for silver nanoparticles generated in NMMO at 70 °C in the shortest time equal to 12 h is comparable to the absorbance for nanoparticles obtained in NMMO at 50 °C but in the longest synthesis time (168 h). This means that by increasing the synthesis temperature it is possible to obtain a similar level of conversion in a shorter period. The absorption spectra of the tested solutions were characterised by different maxima of absorbance depending on the duration of synthesis of nanoparticles. They showed maxima of absorbance at wavelengths: 404 nm (for 72 and 168 h synthesis), 405 nm (for 48 h synthesis) and 406 nm (for the two shortest synthesis times of nanoparticles—12 and 24 h) (Figure 2). Relatively narrow absorption bands may indicate monodispersity.

#### 3.1.4. UV-Vis Results of AgNPs Synthesised at 100 °C

Figure 1d presents the relationship between the absorbance and the wavelength of silver nanoparticles obtained in NMMO at 100 °C in 20 min.

The synthesis of silver nanoparticles in NMMO at 100 °C was carried out at a constant time of 20 min. The UV spectrum for this system showed a maximum absorbance of about 1.54 at 412 nm (Figure 1d). It was noted that the obtained maximum absorbance is comparable with the maximum absorbance obtained for silver nanoparticles synthesised at 70 °C, but with significantly longer synthesis times of 12 and 24 h (Figure 1c). The use of such a high temperature synthesis allowed to shorten significantly the synthesis time, only up to 20 min.

Figure 2 shows the comparison of absorbance values for silver nanoparticles obtained in NMMO depending on temperature and duration of synthesis.

Analysis of the obtained results allows to conclude that at the synthesis temperature of 20 °C the nucleation and growth processes of silver nanoparticles in NMMO are very slow. This makes it necessary to use longer synthesis times. Extending the time of synthesis does not cause a significant increase of the reaction yield. In this case, the absorbance at 0.01 for the 12 h synthesis increases to about 0.3 after extending the time to 168 h. Even the use of such long synthesis times is insufficient to reduce completely the precursor in NMMO. It should be noted that if the process of AgNP synthesis is not completed, the reduction reaction of the remaining amount of the precursor takes place during the dissolution of cellulose. In this case, there is no possibility to control the conditions of nanoparticle formation and, thus, the option to control their morphology does not exist.

When analysing the process of silver nanoparticle synthesis in NMMO at 50 °C, it can be noted that with the increase of the synthesis time, the degree of AgNO3 conversion increases significantly. This is evidenced by the increasing absorbance of the generated silver nanoparticles, which after about 12 h was about 0.3 (Figure 1b). With the extension of the synthesis time, the absorbance rapidly increases to about 1.55 for the longest time of 168 h.

Increasing the temperature to 70 °C contributes to a significant increase in the rate of the synthesis of nanoparticles. Based on the results of the measurements of the absorbance of synthesised nanoparticles as a function of the synthesis time, it can be observed that after a synthesis time of 12 h, the absorbance value is about 1.55. This is comparable with the absorbance of nanoparticles synthesised at 50 °C, but observed after a time equal to 168 h. The use of a synthesis temperature of 70 °C contributes to a small increase in the absorbance during the synthesis from about 1.55 to about 1.7 for the longest synthesis time of 168 h. This suggests that the reaction is complete after 12 h of synthesis. The position of the maximum absorbances recorded on the spectra varies with the extension of the synthesis time (the smallest wavelength for the longest synthesis times, i.e., 404 nm, Figure 1e). This indicates the presence of large shares of small silver nanoparticles in the synthesised systems. The synthesis of silver nanoparticles carried out at 100 °C is very fast. It was noted that the maximum absorbance for synthesised nanoparticles under these conditions is about 1.54 in just 20 min and is comparable to the maximum absorbance obtained for the system with nanoparticles synthesised at 70 °C, but in a much longer time equal to 12 and 24 h.

### 3.2. Morphology and Size Distribution of Silver Nanoparticles

Two research methods, i.e., dynamic light scattering (DLS) and transmission electron microscopy (TEM), were used to investigate the size, shape, as well as the size distribution of silver nanoparticles. The analysis of silver nanoparticles using the DLS method included the determination of the size of nanoparticles and their aggregates, their numerical and volume share, and the intensity of scattered light. In the DLS method, the measurement of the size of the nanoparticles is only possible in the solution. Nanoparticles moving freely in the solution may have a tendency to agglomerate, which means that only signals from the agglomerates of nanoparticles on which strong light scattering takes place are recorded. As can be seen from previous experiments, the presence of even a relatively small amount of agglomerates can interfere with the scattering of light on nanoparticles of small diameter [20]. For TEM studies, the observation of nanoparticles occurs in the fibre, which means that the actual image of the “frozen” nanoparticles in the polymer matrix is observed. Therefore, the TEM results can be fruitfully compared with the results obtained with the DLS method [20]. The characterisation of silver nanoparticles using the TEM technique is based on completely different principles from the DLS method, which allows the independent comparison of their results [19]. An additional advantage of the TEM method is the possibility of direct observation of objects, as well as the estimation of the shape and distribution of silver nanoparticles in the polymer matrix of the fibre.

#### 3.2.1. Determination of the Size of Silver Nanoparticles Using Dynamic Light Scattering (DLS)

The particle size studies were carried out in aqueous NMMO solutions containing silver nanoparticle dispersions (directly after the synthesis of AgNPs).

##### DLS Results of AgNPs Synthesised at 20 °C

The characteristics of AgNP dispersion—AgNPs synthesised at 20 °C at various times in an NMMO solution—are presented in Table 1.

According to the assumptions of the DLS theory, the results of analyses were divided into three fractions depending on the size of the particles in the tested solution. Detailed analysis of DLS results and the relevant research methodology have been included in previous works [19,21].

Based on the obtained results, it can be concluded that the synthesis of silver nanoparticles in NMMO at 20 °C at different times allows the generation of nanoparticles with diameters of 2–40 nm, as shown in Table 1. Based on the UV-Vis results, in these conditions, the reaction of synthesis nanoparticles is not completed, so the next stage of the reaction takes place during the process of cellulose dissolution, i.e., in conditions of changing temperature and concentration of reagents. The DLS measurements of AgNPs diameters of nanoparticles synthesised in NMMO at the temperature of 20 °C are difficult, mainly due to the elliptical shape of the nanoparticles. Reliable measurements of nanoparticle size by DLS method are possible only on spherical objects. Since the yield of reduction carried out at 20 °C is relatively low, only a small number of nanoparticles could be estimated by DLS method. As it was written above, most of the nanoparticles are generated in the laboratory kneader (during the spinning dope preparation) in the presence of cellulose, antioxidant and continuously elevating temperature. Producing AgNPs at relatively low temperatures requires extremely long reaction times what, from a practical point of view, is not favourable.

##### DLS Results of AgNPs Synthesised at 50 °C

The characteristics of AgNPs synthesised at 50 °C at various times in an NMMO solution are presented in Table 2.

The results obtained by the DLS method for AgNP dispersion in NMMO solutions show that nanoparticles synthesised at 50 °C allow to generate nanoparticles in diameters ranging from about 1.2 nm to about 40 nm and agglomerates of about 100–2000 nm. The largest agglomerates (share below 0.1%) are present only in the solution NMMO-Ag/50/12, i.e., in the case of the shortest 12 h synthesis of nanoparticles in NMMO (Table 2). In Table 2, it can be seen that the highest numerical and volume fraction (about 100%) belongs to the smallest nanoparticles of diameters in the range of about 2–5 nm. The intensity of the scattered light on the small particles is still less compared to the intensity of light scattered by the very few but large agglomerates of particles formed in the solutions.

##### DLS Results of AgNPs Synthesised at 70 °C

The characteristics of AgNP dispersion are presented in Table 3.

The results obtained by the DLS method for the dispersion of AgNPs in NMMO show that for 12–24 h synthesis, light scattering occurs only on agglomerates of nanoparticles. The size of those agglomerates ranges from about 50 nm to about 70 nm. For AgNPs synthesised in at 70 °C the presence of fraction 1 is not observed. Fraction 2 about 6–32 nm is observed for synthesis times 48–168 h. Fraction 3 contains agglomerates of nanoparticles whose diameters in an NMMO system with nanoparticles obtained during 48, 72 and 168 h synthesis are about 100 nm.

##### DLS Results of AgNPs Synthesised at 100 °C

The characteristics of AgNP dispersion incubated at 100 °C in 20 min are presented in Table 4.

The DLS results show that the dispersion of silver nanoparticles obtained at 100 °C in 20 min contains only very small particles with dimensions of about 2 nm.

#### 3.2.2. Estimation of the Size and Shape of Silver Nanoparticles in Cellulose Fibres Using the TEM Method

The TEM method allows to characterise more precisely the generated silver nanoparticles, closed in the polymer matrix of cellulose fibres. The created images allowed to observe the size distribution and shape of the nanoparticles and their agglomerates obtained in fibres, and their distribution in the polymer matrix of fibres (images in the “bright field”). The TEM images analysis allowed to determine the histograms of the size distribution of nanoparticles (these histograms were developed on the basis of the count of nanoparticles in over a dozen photos). For the histograms, the average size of the particles, the minimum and maximum diameters (Dmin, Dmax), as well as the average aspect of elliptical particles (W) and eccentricity of ellipse (e) were calculated.

Selected, typical TEM images and histograms of the size distribution of silver nanoparticles obtained at 20 °C for different times of synthesis and introduced into cellulose fibres are shown in the figures below.

In the case of the analysis of the diameters of nanoparticles synthesised at 20 °C, the only reliable method to observe the nanoparticles closed in the fibre matrix was using the TEM technique. This is due to the fact that nanoparticles were generated in various conditions, mainly in direct contact with cellulose pulp and in the elevating temperature, as shown by UV-Vis (Figure 2). The TEM results should not be compared with the results obtained for the small amount of nanoparticles in NMMO solution, which were shown by DLS method. Based on the obtained results, it can be concluded that the synthesis of silver nanoparticles in NMMO at 20 °C at different times allows the generation of nanoparticles with diameters of 2–40 nm, as shown in Table 1. When those nanoparticles were introduced to the fibres, theirs size increased significantly which is typical and characteristic of nanoparticles generated in direct contact with cellulose [19]. In the case of AgNPs generated at relatively low temperatures (20 °C), the TEM results show a mixture of nanoparticles obtained under different conditions.

**Figure 3:**
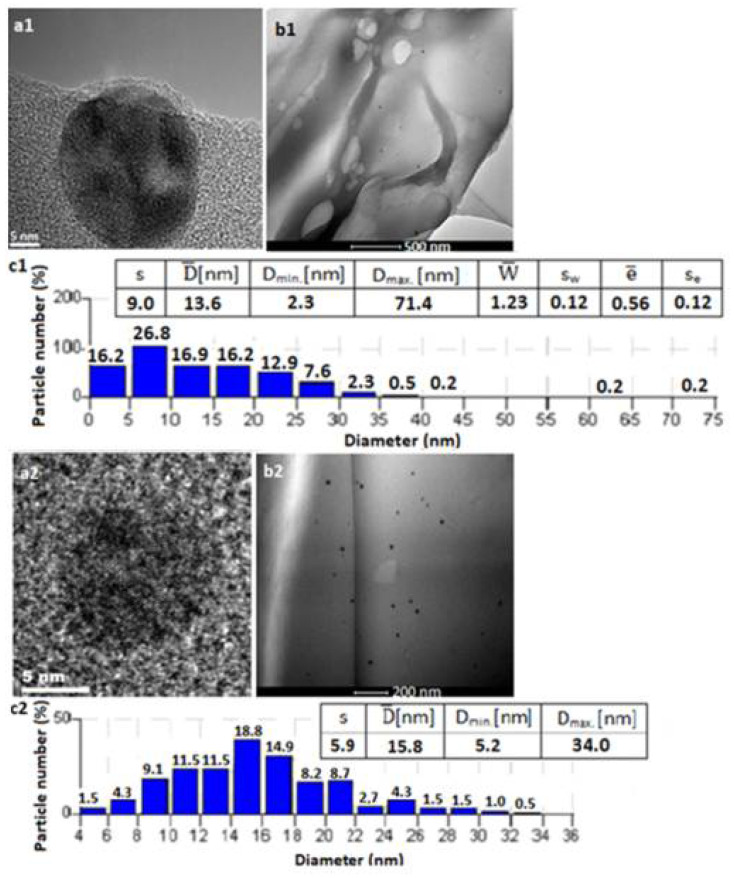
TEM images of silver nanoparticles enclosed in fibres F-Ag/20/72 and F-Ag/20/168: (**a**) under high resolution electron microscopy, (**b**) silver nanoparticles in bright field, (**c**) silver nanoparticles size distributions; where: F-Ag/20/72 (**a1**,**b1**,**c1**) and F-Ag/20/168 (**a2**,**b2**,**c2**).

The TEM images analysis of silver nanoparticles synthesised at 20 °C for different times and then introduced into the fibre matrix shows that with the increase of particle synthesis time, their shape changes from elliptical to spherical. A spherical shape is observed for particles generated in the longest time of synthesis of 168 h. The 48 and 72 h synthesis of nanoparticles allows to obtain the majority of small nanoparticles with diameters from about 2 to 5 nm (Figure 3(a1,b1,c1)). The majority of large particles and their aggregates in the analysed fibre matrix are observed in fibre F-Ag/20/12. This causes large distances between the obtained nanoparticles, as well as their low density in the fibre matrix. The TEM images and histograms made for F-Ag/20/12, F-Ag/20/24 and F-Ag/20/48 have been shown in previous work [20,21]. The TEM images show that in the F-Ag/20/168 fibre modified with nanoparticles synthesised in the longest time (168 h), the size of the nanoparticles ranged between 4 and 34 nm and were relatively evenly distributed in the polymer matrix of the fibre. In addition, the aggregates of particles are not observed in the F-Ag/20/168 matrix (Figure 3). In the analysed fibres, apart from monocrystalline particles, polycrystalline nanoparticles (a1–2) can also be noticed.

**Figure 4:**
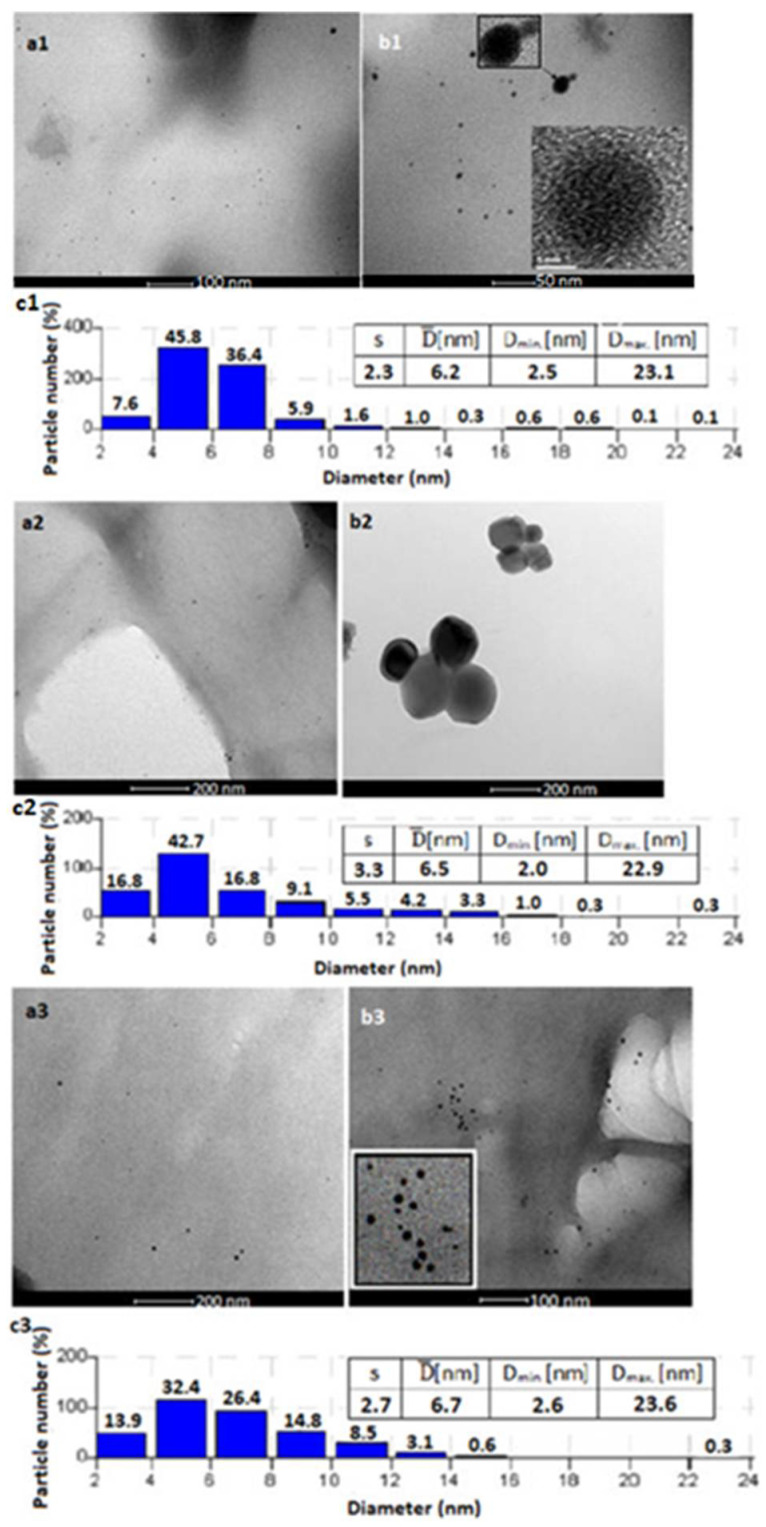
TEM images of silver nanoparticles enclosed in fibres F-Ag/50/24, F-Ag/50/48 and F-Ag/50/72: (**a**,**b**) silver nanoparticles in bright field, (**c**) silver nanoparticle size distributions; where F-Ag/50/24 (**a1**,**b1**,**c1**); F-Ag/50/48 (**a2**,**b2**,**c2**); F-Ag/50/72 (**a3**,**b3**,**c3**).

Typical TEM images and size distributions of silver nanoparticles synthesised in NMMO at 50 °C for different times and introduced into the fibres are shown in Figure 4.

Based on TEM images of modified fibres, it can be concluded that AgNP synthesis at 50 °C allows the formation of small monocrystalline particles (diameters from 2 to 6 nm), as well as very small particle aggregates which correlates with the diameters of nanoparticles, which were observed in the NMMO dispersion by the DLS method (Table 2). It was noticed that with the extension of AgNP synthesis time (from 12 to 48 h), the proportion of small particles with diameters of 4–6 nm (from about 2% to about 50%) increased which is also confirmed by the DLS results of diameter of silver nanoparticles in NMMO dispersion. Large aggregates of nanoparticles (from about 100 to 400 nm) are observed in the fibre matrix. The histograms show that in fibres F-Ag/50/72 and F-Ag/50/168 smaller amounts of tiny particles in the range of 4–6 nm (about 30%) are observed than in fibres F-Ag/50/24 and F-Ag/50/48. Only very small aggregates of particles with a size of just a few nanometres can be observed in the TEM images.

**Figure 5:**
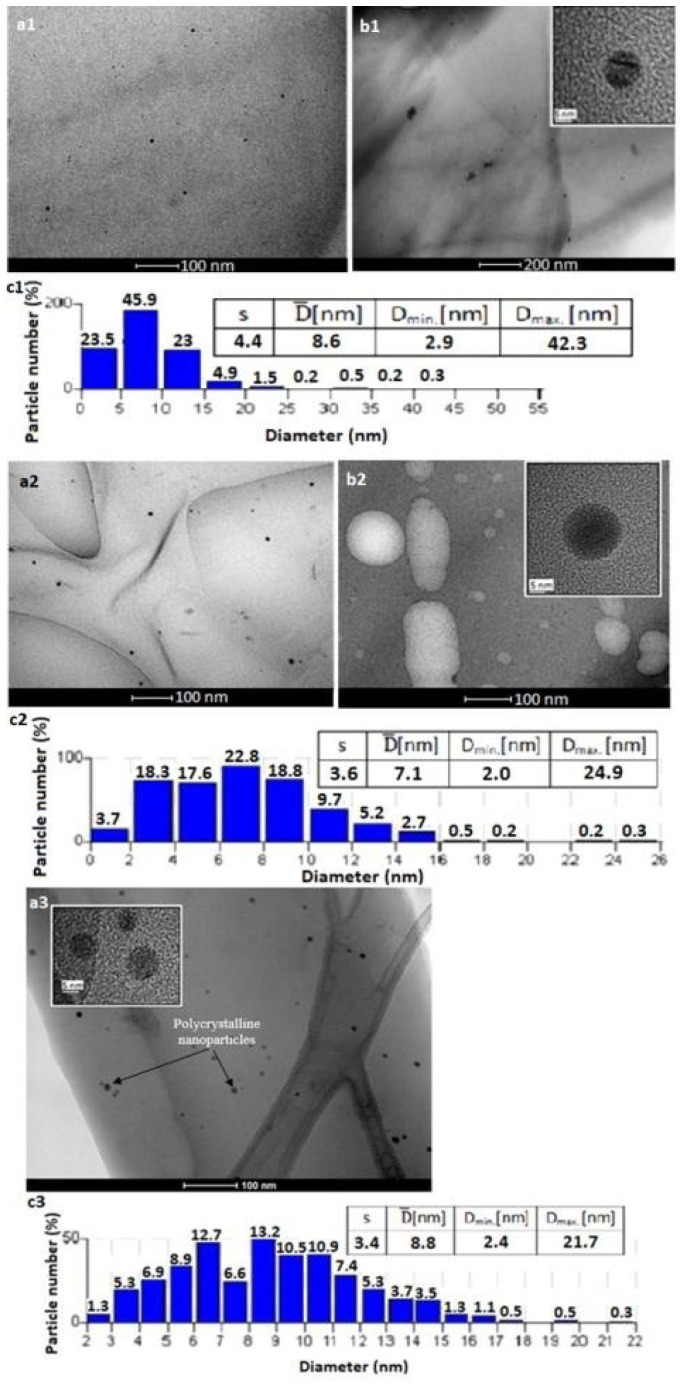
TEM images of silver nanoparticles enclosed in fibres F-Ag/70/12, F-Ag/70/24 and F-Ag/70/168: (**a**,**b**) silver nanoparticles in bright field, (**c**) silver nanoparticle size distributions; where F-Ag/70/12 (**a1**,**b1**,**c1**); F-Ag/70/24 (**a2**,**b2**,**c2**); F-Ag/70/168 (**a3**,**c3**).

Selected TEM images and size distributions of silver nanoparticles synthesised in NMMO at 70 °C for different times and introduced into the fibres are shown in Figure 5.

The synthesis of silver nanoparticles at 70 °C allows for such modification of cellulose fibres, whereby the particles are statistically distributed in the fibres matrix, in the range of 2–42 nm. The highest numerical share of particles (Figure 5(a1–c1)), with the majority of particles which have a radius diameter ranging from 2 to 5 nm (about 90%), is clearly visible in TEM images of fibre F-Ag/70/12. A small proportion of nanoparticle aggregates and agglomerates can be noted in this fibre. In the fibres modified with AgNPs synthesised in longer times, i.e., from 24 to 168 h, smaller amounts of particles are observed. In fibre F-Ag/70/168 modified with nanoparticles synthesised at 70 °C for the longest time (168 h), the smallest amount of particles can be observed (Figure 5(c3)). This is related to the presence of a majority of particles with relatively large diameters in the fibre matrix (mainly between 6 and 13 nm), as well as their aggregates (between 20 and 100 nm) in comparison to the size of nanoparticles in other fibres. In the obtained fibres modified with nanoparticles synthesised at 70 °C, polycrystalline particles (a3) can also be observed.

**Figure 6:**
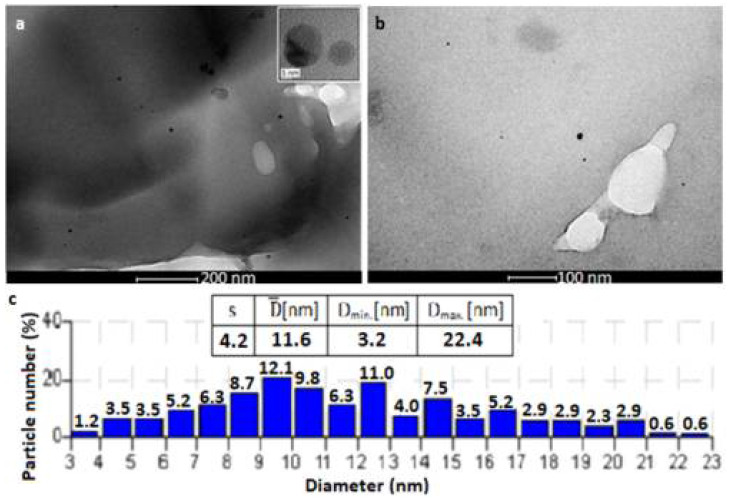
TEM images of silver nanoparticles enclosed in fibres F-Ag/100/0.33: (**a**,**b**) silver nanoparticles in bright field, (**c**) silver nanoparticle size distributions.

It can be clearly observed that both the temperature increase to 70 °C and the long synthesis time of silver nanoparticles in NMMO adversely affect the process of studying nanoparticle size using the DLS method. Comparing the obtained results of nanoparticle diameters in NMMO with the diameters of nanoparticles closed in cellulose fibres, it can be noticed that the analysis of nanoparticles using the DLS method shows mainly aggregates of silver nanoparticles that are formed in NMMO under such synthesis conditions and which can also be seen in the fibres in the TEM images (Figure 5(b1)). However, the TEM research showed the participation of nanoparticle aggregates in a small proportion, with a predominance of nanoparticles with dimensions at the level of 2–13 nm. It was noticed that even a short time of conducting the DLS test favours the formation of agglomerates of silver nanoparticles generated in such conditions. Selected TEM images and size distributions of silver nanoparticles synthesised in NMMO at 100 °C over 20 min and introduced into the fibres are shown in Figure 6.

In fibre F-Ag/100/0.33 modified with AgNPs synthesised at 100 °C in 20 min, mainly polycrystalline particles ranging between 3 and 22 nm with spherical shape were obtained and this is visible in the fibre matrix. A sizeable number (70%) of quite large nanoparticles, between 7 and 15 nm, with a very small amount of aggregates (about 30 nm) can be observed in the fibre matrix. In the case of the synthesis of nanoparticles at the highest temperature and the shortest time, DLS studies of the NMMO-AgNP dispersion showed the participation of only small nanoparticles with a diameter of 3 nm, the share of which is visible in the fibre matrix. Nevertheless, it can be concluded that the analysis of fibres by the TEM method is more precise and showed that there are also nanoparticles of larger dimensions in the fibre, and the largest proportions of nanoparticles have diameters of 7–15 nm. The results of measurements of the size of nanoparticles and particle agglomerates carried out by TEM methods are compared in Table 5.

The applied synthesis conditions, temperature and time, play an important role in the process of obtaining silver nanoparticles. Increasing the temperature of synthesis causes an increase in the precursor reduction rate, which increases the nucleation rate and, as a result, yields large (up to about 10 nm) and extremely small (below 2 nm) spherical nanoparticles. Increasing the reaction temperature shortens the synthesis time significantly.

The high degree of conversion of the precursor eliminates extremely difficult to control factors during cellulose dissolution, i.e., the temperature and composition of the mixture. The mentioned factors change during the cellulose dissolution process.

An important parameter affecting the synthesis of silver nanoparticles in NMMO is time. For reaction temperature equal to 20 °C only a significant prolongation of the AgNO_3_ reduction time is sufficient to increase the rate of silver nitrate conversion and produce more small silver nanoparticles.

The application of higher temperatures shortens significantly the time of synthesis. It has been noted that in the synthesis of nanoparticles at higher temperatures (50 and 70 °C), prolonging the synthesis time may affect the process of generating nanoparticles. In addition to the formation of very small nanoparticles, aggregates and agglomerates of these particles may be formed (Table 5).

### 3.3. Estimation of the Antibacterial Activity of the Fibres

Due to the fact that the fibres will be intended for potential use for medical purposes, one of the important aims of the research was to determine their antibacterial activity against both Gram-negative and Gram-positive bacteria. The influence of the conditions for the synthesis of silver nanoparticles in NMMO as well as the basic parameters of AgNPs on the antibacterial properties of fibres were estimated.

The obtained results of antibacterial activity selected for testing fibres against *E. coli* (Gram-negative bacteria) and *S. aureus* (Gram-positive bacteria) are presented in Table 6 and Table 7, respectively.

**Figure 7:**
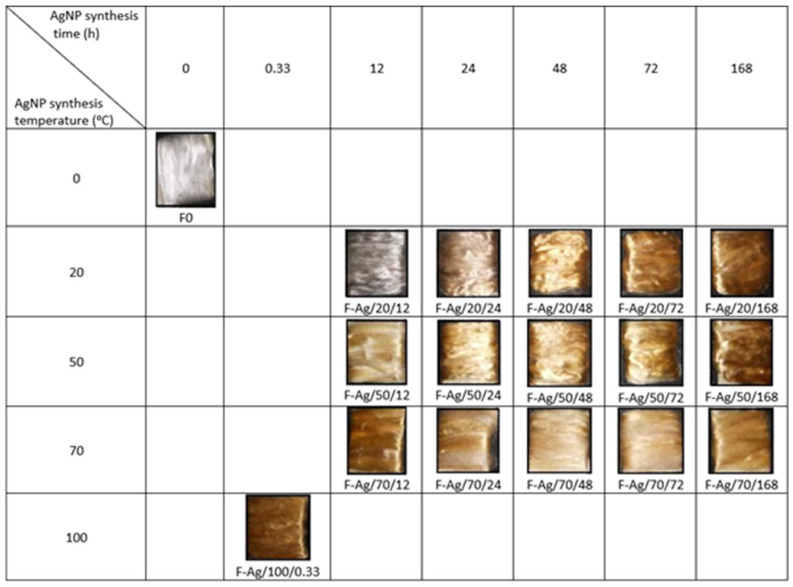
Colour change of fibres depending on AgNP synthesis parameters.

The bioactive fibres obtained different colours depending on the temperature and time of silver nanoparticle synthesis (Figure 7). Noteworthy is the fact that when testing the antibacterial properties of the fibres, it was observed that the fibres modified with nanoparticles change their colour after contact with bacterial cells. For example, a change of colour from golden to cream of bioactive fibres after contact with bacterial cells was observed.

Based on the results shown in Table 6 and Table 7, the antibacterial activity of fibre F-Ag/20/12 is very low. According to some researchers, such a low values of the bactericidal coefficient (L) and bacteriostatic (S) can suggest even a lack of bacteriostatic and bactericidal activity [23,24]. F-Ag/20/12 fibre contains silver nanoparticles of an elliptical shape (bean-shaped grains) and diameters mainly at the level of about 20 nm. Those AgNPs tend to form large aggregates what was proved by DLS and TEM analysis. In case of the F-Ag/20/12 fibre, low antibacterial activity can be explained by the presence of relatively large nanoparticles and their aggregates. This results in relatively large distances between the AgNPs in the fibre matrix and their relatively small surface area in contact with bacteria. The low antibacterial activity excludes this fibre from a group of fibres suitable for medical applications. The F-Ag/50/48 fibre shows only bacteriostatic activity against both types of bacteria. The fibres contain AgNPs with diameters in the range of 2–24 nm, and large aggregates of bigger nanoparticles at the level of about 100–400 nm (TEM analysis, Figure 4(b2)). Bacteriostatic and bactericidal activity against both types of bacteria are rather specific to fibres containing AgNPs synthesised over longer times. Fibres modified with silver nanoparticles synthesised at 20 °C during 24 and 48 h showed very good bacteriostatic and bactericidal activity against *E. coli* bacteria, while bactericidal and bacteriostatic properties against *S. aureus* bacteria have lower values. The good antibacterial activity of the F-Ag/20/24 fibre is due to the fact that no nanoparticle aggregates were found in the fibre. According to the TEM and DLS estimation the share of nanoparticles with dimensions ranging from about 8 to 30 nm is high. In case of the F-Ag/20/48 fiber a very high proportion of very small silver nanoparticles at a level of about 2–5 nm was observed. It seems that the presence of small AgNPs with almost no aggregates has an impact on the satisfactory antibacterial properties of the fibres [20]. The highest values of bacteriostatic and bactericidal effectiveness against both Gram-negative and Gram-positive bacteria were found in F-Ag/50/12, F-Ag/50/24, F-Ag/70/12, F-Ag/70/48 and F-Ag/100/0.33 fibres. Bacteriostatic and bactericidal effectiveness for these fibres are high, which means that these fibres showed very good antibacterial properties against both types of bacteria. It can be noticed that the F-Ag/70/24 fibre showed high antibacterial properties against *E. coli* and lower against *S. aureus*, especially regarding their bactericidal properties. A weaker bactericidal activity of fibres results from the presence of agglomerates in their matrix (Figure 5(a2)). In other fibres showing high activity against both *E. coli* and *S. aureus* bacteria, small silver nanoparticles (2–15 nm) are observed, which due to their large active surface have excellent cell deactivating properties of bacteria.

Based on the tests of antibacterial activity of selected fibres modified with silver nanoparticles, it can be stated that the conditions of silver nanoparticles synthesis on the bioactive properties of the fibres are significant.

## 4. Conclusions

Cellulose fibres modified with AgNPs synthesised in the NMMO system were obtained. It can be concluded that from the point of view of the morphology of nanoparticles and their distribution in the polymer matrix of fibres, particle synthesis under high-temperature conditions of 70–100 °C is preferred. It seems that the optimal synthesis conditions are at 100 °C and over 20 min. The results of the antibacterial properties of the fibres show that the conditions of a high-temperature of nanoparticle synthesis, especially 100 °C, influence the obtaining of silver nanoparticles with optimal parameters (3–22 nm and very small aggregates) in the fibre and allow obtaining fibres with strong bioactive properties. Good effects can also be obtained at 50 °C. In this case, a much longer synthesis time is required (over 72 h) to completely convert the precursor.

Based on the presented work, the hypothesis that it is possible to control the process of synthesis of AgNPs using NMMO as a reducing system was confirmed. The estimation of the basic parameters of AgNPs enclosed in fibres, i.e., their diameter and shape, as well as the size and amount of their aggregates and agglomerates, is essential in the selection of the best conditions for AgNP synthesis. It is possible to estimate the influence of reaction conditions on the mentioned AgNP parameters and their distribution in the matrix of fibres and, as a result, understanding the synthesis conditions allowed to control the process in such a way that the nanocomposite bioactive cellulose fibres were obtained in optimal conditions. These fibres contain the lowest possible concentration of AgNPs (ensuring antibacterial properties), which are evenly distributed in the cellulose matrix with the smallest possible amount of aggregates and agglomerates. The synthesis of nanoparticles with the simultaneous production of fibres allows obtaining an innovative antibacterial and antiviral material with a high application potential, which can be used to obtain, for example, masks, gloves, hygiene materials, dressings, etc.

## Figures and Tables

**Table 1:** Results of the DLS analysis of AgNPs synthesised in an NMMO system at 20 °C.

Sample	Fraction No.	Volume Weighting		Number Weighting		Intensity Weighting	
		Diameter (nm)	Percentage (%)	Diameter (nm)	Percentage (%)	Diameter (nm)	Percentage (%)
NMMO	1	423.7	100	410.5	100	408.4	100
NMMO-Ag/20/12	1	2.3	98.4	2.2	100	2.4	15.0
	2	15.2	1.6	14.5	0.0	17.5	85.0
	3	----	----	----	----	----	----
NMMO-Ag/20/24	1	2.5	97.4	2.5	99.9	2.3	0.6
	2	44.2	2.6	42.0	0.1	48.7	99.4
	3	----	----	----	----	----	----
NMMO-Ag/20/48	1	3.2	97.9	3.0	99.8	3.8	4.9
	2	32.4	2.1	30.7	0.2	35.8	95.1
	3	----	----	----	----	----	----
NMMO-Ag/20/72	1	3.6	93.5	3.4	98.4	4.0	6.7
	2	15.2	6.3	14.8	1.6	16.7	31.4
	3	70.2	0.1	68.7	<0.1	83.1	62.0
NMMO-Ag/20/168	1	2.5	84.8	2.5	95.2	2.4	4.2
	2	10.0	15.1	9.5	4.8	11.2	54.1
	3	52.6	0.1	----	----	58.0	41.7

**Table 2:** Results of the DLS analysis of AgNPs synthesised in an NMMO system at 50 °C.

Sample	Fraction No.	Volume Weighting		Number Weighting		Intensity Weighting	
		Diameter (nm)	Percentage (%)	Diameter (nm)	Percentage (%)	Diameter (nm)	Percentage (%)
NMMO	1	423.7	100	410.5	100	408.4	100
NMMO-Ag/50/12	1	2.5	99.9	2.4	100	2.3	1.5
	2	106.3	0.1	102.9	<0.1	121.8	77.0
	3	1929.3	<0.1	1920.5	<0.1	1920.5	21.5
NMMO-Ag/50/24	1	1.3	55.0	1.2	0.8	1.2	0.8
	2	5.4	45.0	6.0	50.4	6.0	50.4
	3	100.8	<0.1	107.3	48.8	107.3	48.8
NMMO-Ag/50/48	1	5.0	99.9	4.9	100.0	5.6	60.6
	2	40.3	0.1	39.7	<0.1	48.6	39.4
	3	----	----	----	----	----	----
NMMO-Ag/50/72	1	2.5	100.0	2.3	100.0	2.7	29.9
	2	204.4	<0.1	200.0	<0.1	2333.3	70.1
	3	----	----	----	----	----	----
NMMO-Ag/50/168	1	1.5	78.8	1.5	90.5	1.6	24.9
	2	3.7	21.2	3.6	9.5	4.0	75.1
	3	41.7	<0.1	41.0	<0.1	----	----

**Table 3:** Results of the DLS analysis of AgNPs synthesised in an NMMO system at 70 °C.

Sample	Fraction No.	Volume Weighting		Number Weighting		Intensity Weighting	
		Diameter (nm)	Percentage (%)	Diameter (nm)	Percentage (%)	Diameter (nm)	Percentage (%)
NMMO	1	423.7	100	410.5	100	408.4	100
NMMO-Ag/70/12	1	----	----	----	----	----	----
	2	----	----	----	----	----	----
	3	71.7	80.7	69.3	98.1	82.2	43.9
NMMO-Ag/70/24	1	----	----	----	----	----	----
	2	-	-	-	-	-	-
	3	51.2	91.3	50.4	99.0	55.0	33.0
NMMO-Ag/70/48	1	----	----	----	----	----	----
	2	32.2	77.6	31.0	91.4	----	----
	3	111.4	22.4	104.9	8.6	122.0	100.0
NMMO-Ag/70/72	1	-	-	-	-	-	-
	2	25.2	35.1	24.2	92.0	26.9	0.8
	3	120.9	12.9	116.8	6.3	139.1	28.4
NMMO-Ag/70/168	1	-	-	-	-	-	-
	2	6.2	97.8	6.2	100.0	5.8	1.3
	3	101.2	0.5	97.9	<0.1	115.9	27.0

**Table 4:** Results of the DLS analysis of AgNPs synthesised in an NMMO system at 100 °C.

Sample	Fraction No.	Volume Weighting		Number Weighting		Intensity Weighting	
		Diameter (nm)	Percentage (%)	Diameter (nm)	Percentage (%)	Diameter (nm)	Percentage (%)
NMMO	1	423.7	100	410.5	100	408.4	100
NMMO-Ag/100/0.33	1	3.0	100	3.0	100	3.0	100
	2	----	----	----	----	----	----
	3	----	----	----	----	----	----

**Table 5:** Comparison of selected TEM results for the fibres modified with AgNPs generated in the shortest possible synthesis time, in which the highest possible conversion of the precursor at the used temperatures has been achieved.

T (°C)	t (h)	TEM			
		Diameter (nm)	Diameters of Nanoparticles with a Share >70%	Sizes of Agglomerates and Aggregates (nm)	Average Size of Nanoparticles (nm)
20	168	4–34	8–22	-	16
50	168	2–20	2–9	10–100	7
70	12	2–42	3–15	~15–100	9
100	0.33	3–22	7–15	~40	12

**Table 6:** Results of tests on the antibacterial activity of the modified fibres against *E. coli*.

Sample	Time (h)	Number of Bacteria (jtk/pr)	Bacteriostatic Effectiveness (S)	Bactericidal Effectiveness (L)	Antibacterial Activity
Reference sample	0	2.1 × 10^4^	–	–	–
Reference sample	24	1.4 × 10^8^	–	–	3.8
F-Ag/20/12	24	1.8 × 10^6^	1.8	−2.0	Only bacteriostatic
F-Ag/20/24	24	<20	6.8	3.0	Strong
F-Ag/20/48	24	<20	7.0	3.7	Strong
F-Ag/50/12	24	<20	6.9	2.7	Significant
F-Ag/50/24	24	<20	6.9	3.0	Strong
F-Ag/50/48	24	1.8 × 10^5^	2.8	−1.0	Only bacteriostatic
F-Ag/70/12	24	<20	6.9	2.7	Significant
F-Ag/70/24	24	<20	6.9	3.0	Strong
F-Ag/70/48	24	<20	6.7	3.2	Strong
F-Ag/100/0.33	24	<20	6.9	3.0	Strong

**Table 7:** Results of tests on the antibacterial activity of the modified fibres against *S. aureus*.

Sample	Time (h)	Number of Bacteria (jtk/pr)	Bacteriostatic Effectiveness (S)	Bactericidal Effectiveness (L)	Antibacterial Activity
Reference sample	0	3.7 × 10^4^	–	–	–
Reference sample	24	7.7 × 10^6^	–	–	3.8
F-Ag/20/12	24	9.5 × 10^4^	1.9	−0.4	Only bacteriostatic
F-Ag/20/24	24	<20	4.6	2.3	Significant
F-Ag/20/48	24	1.9 × 10^2^	4.9	2.4	Significant
F-Ag/50/12	24	<20	5.7	3.5	Strong
F-Ag/50/24	24	<20	5.7	3.3	Strong
F-Ag/50/48	24	6.2 × 10^4^	2.1	−0.2	Only bacteriostatic
F-Ag/70/12	24	<20	2.9	1.1	Negligible
F-Ag/70/24	24	<20	5.7	3.3	Strong
F-Ag/70/48	24	<20	5.7	3.3	Strong
F-Ag/100/0.33	24	<20	5.7	3.3	Strong

## Data Availability

Data sharing is not applicable to this article.
